# Physicians’ perceived barriers to management of sexually transmitted infections in Vietnam

**DOI:** 10.1186/1471-2458-14-1133

**Published:** 2014-11-04

**Authors:** Khoi Do, Victor Minichiello, Rafat Hussain, Asaduzzaman Khan

**Affiliations:** School of Rural Medicine, University of New England, Armidale, NSW 2350 Australia; School of Medicine and Public Health, University of Newcastle & Emeritus Professor, University of New England, Armidale, NSW 2350 Australia; School of Health and Rehabilitation Sciences, The University of Queensland, Armidale, NSW 2350 Australia

**Keywords:** Sexually transmitted infection, Physician, Barrier, Vietnam

## Abstract

**Background:**

Sexually transmitted infections (STIs) are a public health problem in Vietnam with sub-optimal care in medical practice. Identifying practitioners’ perceived barriers to STI care is important to improve care for patients with STIs.

**Methods:**

A cross-sectional survey was conducted among 451 physicians. These physicians were dermatology and venereology (D&V) doctors, obstetrical/gynaecological (Ob/Gyn) doctors, general practitioners, and assistant doctors working in health facilities at provincial, district and communal levels in three provinces in Vietnam.

**Results:**

Almost all (99%) respondents mentioned at least one barrier to STI care. The barriers were “lack of STI training” (57%), “lack of professional resources” (41%), “lack of time” (38%), “lack of reimbursement” (21%), “lack of privacy/confidentiality” (17%), “lack of counselling” (15%), and “not the role of primary care provider” (7%). Multivariable logistic regression analysis showed that “lack of professional resources” was associated with respondents being in medical practice for ten years or under (vs. 11–20 years), and working at district or communal health facilities (vs. provincial facilities); “lack of time” were associated with respondents being female, seeing more than 30 patients a week (vs. <15 patients/week); and “lack of privacy/confidentiality” was associated with physicians’ seeing more than 30 patients a week (vs. <15 patients/week).

**Conclusion:**

The study has identified several barriers to STI care in medical practice in Vietnam. Results of the study can be used to improve areas in STI care including policy and practice implications.

## Background

Sexually transmitted infections (STIs) are a major public health problem globally [1]. Without proper treatment, STIs can cause serious medical and psychological consequences and ultimately death [2]. In addition, STIs can facilitate transmission of HIV. It has been estimated that ulcerative STIs can increase HIV transmission risk by 10–50 times for male-to-female, and by 50–300 times for female-to-male transmission, while non-ulcerative STIs can increase HIV transmission risk by 2–5 times [3]. Two Cochrane reviews [4, 5] found that although there is limited evidence for better STI control measures as a prevention strategy for HIV, there are, nonetheless, other compelling reasons for improving STI management [4, 5]. Important among these are providing an increased quality of service, and changing sexual behaviour such as promoting condom use and thereby having an impact on reducing the incidence of some of the STIs as well as HIV [6–8].

In Vietnam, the reported number of STIs increased more than six-fold between 1996 and 2010 [9]. It had been estimated that there were one million new STI cases in Vietnam annually [10, 11]. Similarly, the reported number of HIV-positive cases increased almost 40 times between 1996 and 2013 [12, 13]. Since 1986, Vietnam abandoned the central planning model of socialism and changed to a market oriented economy, known as Doi Moi. Under this renovation, the economy and the society have undergone significant changes such as decollectivization in agricultural production, development of private sector and foreign investment [14]. While the renovation has been successful in economic development, impacts on other aspects of society should be noted including unemployment, reduced availability of public health, increased inequality, widening income gap, increasing rural to urban migration [15, 16]. These impacts have disproportionately affected women and some of them resorted to prostitution as a means to survive [17, 18]. The emergence of sex work, together with inconsistent condom use, might have contributed to the increase in STI cases in Vietnam [19–21]. As for the health services, inconsistencies in physicians’ practice such as insufficient risk assessment, improper diagnosis and treatment, inefficient behavioural change counselling could also be linked to the increase of STI cases [22–24].

Healthcare providers play a crucial role in reducing the burden of STI/HIV by providing effective STI prevention and case management [25]. However, while inadequacies in involvement of practitioners in offering optimal care for STI have been reported [26–28], it also needs to be acknowledged that the health care system and, how it is financed, influences the delivery of health services. Available data from Vietnam suggest there are issues in STI management such as the tendency to over-diagnose reproductive tract infections (RTIs) by clinicians and laboratory technicians [29], insufficient knowledge of STIs by physicians [22], and some patients felt that they were not being treated with respect such as being shouted at or scold by physicians and not satisfied with the services they receive at health facilities [30, 31]. A better understanding of physicians’ views on barriers in management of STIs is an important area of research to inform current policies and programmes in Vietnam. This study aimed to determine physicians’ perception of barriers to STI services and factors related to such barriers.

## Methods

A cross-sectional self-administered survey of 451 physicians working in three provinces (Quang Ninh, An Giang and Can Tho) of Vietnam was conducted in September-November 2010. These provinces were identified as having a more serious STI/HIV problem in Vietnam [32]. Potential respondents were physicians in clinical practice at health facilities at provincial, district and communal levels. To be eligible, the respondents needed to be dermatology and venereology (D&V) doctors, obstetric/gynaecological (Ob/Gyn) doctors, general practitioners, or assistant doctors. As the number of D&V doctors per province was small, all available D&V doctors were included in the survey. Non D&V physicians (Ob/Gyn doctors, general practitioners, or assistant doctors) were selected using a multi-stage sampling method where ten districts among the 29 districts in the three provinces were selected using simple random sampling and subsequently a systematic random sampling was conducted among the 10 selected districts to select the required number of physicians [33].

Vietnam is divided into provinces. The provinces are further divided into town and districts. Towns and districts are subsequently divided into communes [34]. The national authority in health is the Ministry of Health responsible for healthcare administration. The provincial health bureaus are under Ministry of Health in terms of technical management and under Provincial People’s Committee in relation to governmental management. The primary level in healthcare network is district health centres and communal health stations [35]. In Vietnam, STI services are provided at general practice and D&V specialised facilities, at public and private facilities. Patients can choose to go directly to D&V facilities first without being referred from general practitioners.

Briefing meetings took place between the researcher and the potential respondents to introduce the survey to them and discuss any questions they might have. Respondents received an information pack containing a background information sheet, informed consent form, a copy of the survey questionnaire, and a return envelope. The respondents were given the option to complete the questionnaire immediately after the briefing meetings or at a later time. A financial incentive of AU$5 was provided to all respondents for their time spent on completing the survey. Respondents who did not return the completed questionnaire on time received three reminders, in two-week interval, from either the researcher or from a staff member of the local health service. Full ethics approval for undertaking the study was granted by the Human Research Ethics Committee of the University of New England, Australia (HE10/148). Additional information about survey arrangement and health setting in Vietnam have been presented in a previously published paper [24].

### Statistical analysis

Data for this paper were sourced from a sub-section of the questionnaire items dealing with management of STIs, and, in particular, perceived barriers to STI care. Perceived barriers to STI care were considered as outcome variables of interest for the present paper. The list of barriers include: “lack of time”, “lack of reimbursement”, “lack of privacy/confidentiality”, “lack of counselling”, “lack of professional resources”, “lack of training in STI” were selected to be included in the questionnaire, based on literature review [36–39]. The independent variables considered in this analysis were physicians’ age, sex, medical degree, training in STIs, training in communication with patients, duration of medical practice, average weekly client volume, STI diagnoses in the month prior to the survey, and place of main practice. These nine independent variables have been reported to be associated with STI knowledge and practice of physicians [36, 40–46]. Independent variables found to be associated with outcome variables at p ≤0.2 (using Chi-square test) at univariable level were included in the base model of multivariable logistic regression [47]. In addition, “clinically plausible” relationships between the barriers were also examined.

Assumptions for logistic regression were checked to ensure that the fitted model was appropriate for the collected data. Assumptions for logistic regression included absence of multicollinearity and of outliers in the solution, and adequate sample size [48, 49]. Variance Inflation Factor (VIF) value over 5 was used to determine multicollinearity [50]. Outliers were checked by examination of residuals. Cases with a standardised residual value over ±2.5 were removed from analysis [49]. The regression models were examined for goodness of fit using the Hosmer–Lemeshow Goodness of fit test; a good fit was indicated by a p-value greater than 0.05. The Wald test was used to evaluate the importance of each of the independent variables. An independent variable was considered significantly associated with the outcome variable of interest if the associated p-value was less than 0.05 [49]. The multivariable logistic regression provided estimates of adjusted odds ratios and their 95% confidence intervals (CI) for respondents’ characteristics associated with perceived barriers to STI care. All data analysis was performed using SPSS, version 20 [51].

## Results

### Respondent characteristics

Of the physicians who participated in the study (n = 451), two-thirds (68.7%) were females and one-third were males (31.3%). While 10% of the participants were D&V doctors, the remaining (90%) were general physicians, Ob/Gyn doctors and assistant doctors. A quarter (26.4%) had been in medical practice for ten years or less, over one-third had been in practice for 11–20 years (34.4%), and the rest (39.2%) had been in practice for over 20 years. Almost half (46.6%) of the respondents had an average weekly patient volume of 15 or fewer; a quarter (24.8%) of the sample saw 16–30 patients and over a quarter (28.6%) had more than 30 patients a week. Regarding the place of main practice, about half (48.6%) of the respondents worked at communal health facilities, 20.2% of respondents worked at district health facilities and the rest (33%) worked at provincial health facilities (see Table 1).

**Table 1:** **Characteristics of survey respondents**

Respondents’ characteristics	Total N (%)
Sex	
Females	310 (68.7)
Males	141 (31.3)
Age group (in years)	
<40	134 (29.7)
40-50	256 (56.8)
51-60	61 (13.5)
Medical degree	
D&V doctor	47 (10.4)
Ob/Gyn doctor	33 (7.3)
General practitioner	110 (24.4)
Assistant doctor	261 (57.9)
In-service training in STIs	
No	157 (34.8)
Yes	294 (65.2)
In-service patient communication training	
No	204 (45.2)
Yes	247 (54.8)
Duration of medical practice (in years)	
≤10	119 (26.4)
11-20	155 (34.4)
>20	177 (39.2)
Average weekly client volume	
≤15	210 (46.6)
16-30	112 (24.8)
> 30	129 (28.6)
STI diagnoses in the month prior to the survey	
No	137 (30.4)
Yes	314 (69.6)
Place of main practice	
Provincial facilities	149 (33)
District facilities	91 (20.2)
Communal health station	211 (46.8)

### Perceived barriers to STI care

Almost all (98.9%, data not shown) respondents mentioned at least one barrier to STI care. Over one-third (39.5%) of the respondents mentioned one barrier, just less than one-third (29.9%) two barriers, one-fifth (20.6%) mentioned three barriers, and the rest of (8.9%) listed four barriers or more. Among the barriers identified, the most common was “lack of STI training” (56.8%). The second most common barrier was “lack of professional resources” (40.6%), followed by “lack of time” (37.9%). Other barriers included “lack of reimbursement” (21.1%), “lack of privacy/confidentiality” (17.3%), “lack of counselling” (14.6%). As for training needs of the respondents, the most common types of training needed were STI diagnosis (80.9%), counselling of STI patients (61.9%), STI prevention (50.3%), and working with patients who identify themselves as sex workers, drug users, and/or homosexual (31.7%). Distribution of barriers by respondents’ characteristics is presented in Table 2.

**Table 2:** **Perceived barriers for STI care by characteristics of respondents**

Respondent characteristics	Lack of STI training N (%*)	Lack of professional resources N (%*)	Lack of time N (%*)	Lack of reimbursement N (%*)	Lack of privacy/ confidentiality N (%*)	Lack of counselling N (%*)
Sex						
Female	176 (56.8%)	131 (42.3%)	126 (40.6%)	69 (22.3%)	54 (17.4%)	44 (14.2%)
Male	80 (56.7%)	52 (36.9%)	45 (31.9%)	26 (18.4%)	24 (17.0%)	22 (15.6%)
Age group (in years)						
<40	87 (64.9%)	65 (48.5%)	50 (37.3%)	28 (20.9%)	26 (19.4%)	23 (17.2%)
40-50	138 (53.9%)	90 (35.2%)	93 (36.3%)	54 (21.1%)	42 (16.4%)	33 (12.9%)
51-60	31 (50.8%)	28 (45.9%)	28 (45.9%)	13 (21.3%)	10 (16.4%)	10 (16.4%)
Medical degree						
D&V doctor	18 (38.30%)	13 (27.70%)	26 (55.3%)	11 (23.4%)	9 (19.1%)	3 (6.40%)
General practitioner	67 (60.9%)	45 (40.9%)	30 (27.3%)	23 (20.9%)	20 (18.2%)	15 (13.6%)
Ob/Gyn doctor	15 (45.50%)	12 (36.4%)	18 (54.5%)	11 (33.3%)	6 (18.2%)	5 (15.2%)
Assistant doctor	156 (59.8%)	113 (43.3%)	97 (37.2%)	50 (19.2%)	43 (16.5%)	43 (16.5%)
In-service training on STIs						
No	109 (69.4%)	79 (50.3%)	44 (28.0%)	20 (12.7%)	28 (17.8%)	28 (17.8%)
Yes	147 (50.0%)	104 (35.4%)	127 (43.2%)	75 (25.5%)	50 (17.0%)	38 (12.9%)
In-service training on communication						
No	126 (61.8%)	100 (49.0%)	61 (29.9%)	43 (21.1%)	44 (21.6%)	34 (16.7%)
Yes	130 (52.6%)	83 (33.6%)	110 (44.5%)	52 (21.1%)	34 (13.8%)	32 (13.0%)
Duration of medical practice (in years)						
≤10	76 (63.9%)	62 (52.1%)	44 (37.0%)	28 (23.5%)	23 (19.3%)	18 (15.1%)
11-20	93 (60.0%)	49 (31.6%)	47 (30.3%)	28 (18.1%)	31 (20.0%)	22 (14.2%)
>20	87 (49.2%)	72 (40.7%)	80 (45.2%)	39 (22.0%)	24 (13.6%)	26 (14.7%)
Average number of patient seen per week						
≤ 15	121 (57.6%)	88 (41.9%)	65 (31.0%)	38 (18.1%)	30 (14.3%)	25 (11.9%)
16-30	61 (54.5%)	47 (42.0%)	44 (39.3%)	25 (22.3%)	18 (16.1%)	11 (9.8%)
> 30	74 (57.4%)	48 (37.2%)	62 (48.1%)	32 (24.8%)	30 (23.3%)	30 (23.3%)
STI diagnoses prior to survey						
Yes	174 (55.4%)	120 (38.2%)	117 (37.3%)	57 (18.2%)	51 (16.2%)	38 (12.1%)
No	82 (59.9%)	63 (46.0%)	54 (39.4%)	38 (27.7%)	27 (19.7%)	28 (20.4%)
Place of main practice						
Province	77 (51.7%)	44 (29.5%)	79 (53.0%)	29 (19.5%)	29 (19.5%)	23 (15.4%)
District	57 (62.6%)	43 (47.3%)	32 (35.2%)	19 (20.9%)	15 (16.5%)	13 (14.3%)
Commune	122 (57.8%)	96 (45.5%)	60 (28.4%)	47 (22.3%)	34 (16.1%)	30 (14.2%)

*Percentage of respondents who reported perceived barriers for STI care (row %, Response = “No” was omitted).

Assistant doctors were twice more likely than D&V doctors to mention lack of STI training. Compared to respondents who had been in medical practice ten years or less, those who had been in medical practice for 11–20 years were less likely to mention “lack of professional resources”. Respondents working at district and communal health facilities were more likely than those working at provincial level facilities to mention “lack of professional resources”. Male physicians were less likely to mention “lack of time” than females. General practitioners were less likely to mention lack of time than D&V doctors. Respondents seeing more than 30 patients a week were more likely to mention “lack of time” than those seeing 15 or fewer patients a week. Respondents who see more than 30 patients a week were also more likely to mention “lack of privacy/confidentiality” than those who see 15 or fewer patients a week (Table 3). At the bivariate level, lack of counselling was significantly associated with lack of privacy/confidentiality (p < 0.05). Respondents who mentioned lack of counselling were more likely to mention lack of privacy/confidentiality.

**Table 3:** **Logistic regression estimates of factors associated with perceived barriers for STI care**

	Lack of STI training	Lack of professional resources	Lack of time Adj OR	Lack of reimbursement	Lack of privacy/ confidentiality	Lack of counselling
	Adj OR (95% CI)	Adj OR (95% CI)	(95% CI)	Adj OR (95% CI)	Adj OR (95% CI)	Adj OR (95% CI)
Sex: Male (vs. Female)	-	-	0.61 (0.39-0.99)*	-	-	-
Age group (in years)						
40–50 (vs. <40)	NS	NS	-	-	-	-
51-60 (vs. <40)	NS	NS	-	-	-	-
Medical degree						
General practitioner (vs. D&V doctor)	NS	NS	0.43 (0.19-0.99)*	-		-
Assistant doctor (vs. D&V doctor)	2.06 (1.04-4.05)*	NS	NS	-	-	-
Ob/Gyn doctors (vs. D&V doctor)	NS	NS	NS	-	-	-
In-service training on STI: Yes (vs. No)	0.52 (0.33-0.82)*	NS	NS	NS	-	-
In-service training on communication: Yes (vs. No)	NS	0.64 (0.40-0.96)*	NS	-	NS	-
Duration of medical practice (in years)						
11–20 (vs. <10)	NS	0.44 (0.23-0.86)*	NS	-	-	-
>20 (vs. <10)	NS	NS	NS	-	-	-
Average weekly client volume						
16-30 (vs. <15)	-	-	NS	-	NS	-
>30 (vs. <15)	-	-	1.77 (1.06-2.95)*	-	2.26 (1.01-5.06)*	-
STI diagnoses in the month prior to the survey:	-	NS	-	NS	-	-
Yes (vs. No)						
Place of main practice						
District hosp./health centre	-	2.23 (1.24-4.01)*	0.46 (0.25-0.84)*	-	-	-
(vs. provincial facilities)						
Communal health stations	-	2 (1.17-3.43)*	0.43 (0.25-0.74)*	-	-	-
(vs. provincial facilities)						

*p ≤ 0.05 (Wald statistic).

NS: not significant at multivariable logistic regression.

-Independent variables not significantly associated with dependant variable at p ≤ 0.2 at univarible logistic regression and not included in multivariable logistic regression.

## Discussion

The respondents in the study reported a number of barriers to providing STI care in Vietnam. The barriers include a range of issues encountered in clinical interactions with patients such as lack of time, lack of availability of educational resources and training, lack of reimbursement, difficulties in maintaining privacy/confidentiality, and lack of counselling. Training has been identified as one of the major means of improving the quality of STI care [52, 53]. Physicians’ training has been reported to be associated with improved physicians’ comfort with STI patients [43, 54]; lower prejudicial attitude toward people living with HIV/AIDS [55]; improved skill for risk assessment, clinical examination, diagnosis and treatment [52]; more willingness to offer STI testing to patients [36]; improved patient adherence [56] and improved patient’s outcomes [53].

In the present study, over half of the respondents identified lack of STI training as a barrier to STI care. Moreover, the finding that half of the respondents who already had in-service STI training also identified “lack of STI training” as a barrier further underscores the need to review the existing STI training materials and refresher courses. The Law on Medical Examination and Treatment, which came into effect on January 1st, 2011, is expected to create important changes in continuing medical education (CME) in Vietnam. Under the Law, medical practitioners (doctors and assistant doctors) need to be licensed to practise medicine and they need to participate in CME at least once every two years in order to maintain their licence [57]. The CME framework in Vietnam is currently being developed, and therefore it may be timely to advocate for inclusion of an effective STI care module in the CME training programs.

Beyond training issues, another major barrier reported by physicians in the present study was lack of professional resources. Lack of professional resources has been identified as a barrier for physicians to provide healthcare services according to clinical guidelines [38, 58, 59]. Another Vietnamese study of health staff at communal level reported low satisfaction with training materials and technical guidelines (50.4%), access to training and coaching (53.9%), and professional training and coaching (57.6%) [60]. In Vietnam, the National Guidelines for diagnosis and treatment of HIV/AIDS was released in 2009, and the National Guidelines for diagnosis and treatment of STIs was released in November 2013 [61, 62]. These guidelines can be used as the principal reference sources for health staff for information regarding STIs and HIV/AIDS management in Vietnam. In addition to the availability of the guidelines for physicians, training on the new guidelines together with adequate supervision and feedback are needed to ensure physicians’ compliance, which in turn can improve STI services. Perhaps, more intensive specialised training on sexual history taking, doctors assisting patients to feel more comfortable to seek sexual health care and new advances in treatment options should be considered.

Lack of time has been reported as a barrier for physicians’ to adhere to clinical guidelines [38, 58, 59], to conduct sexual history taking and/or discuss with patients about sexual health issues [63–65], to offer HIV test to patients [37, 66]. In the present study, lack of time was a common barrier among the physicians and this was more common among female physicians. This is also consistent with the literature that male physicians tend to spend less time taking a patient’s sexual history or offering STI test to patients [36, 39, 41]. It could also be that female patients tend to prefer female physicians more than male physicians when seeking STI services [67, 68]. In order to help physicians to deal with this issue, time saving tools for physicians need to be made available. The use of job aid such as flipcharts, flowchart may be effective and feasible in developing countries [69–71] and the physicians should be well equipped with these during their interactions with patients. Since sexual history taking can be time-consuming and often not well implemented [24, 41, 72], improvement in this aspect is important to improve quality of STI service and to save physicians’ time. To help physicians save time taking a patient’s sexual history, the use of patient-administered sexual history taking instrument to help screen patients for STI risk has been successfully utilised elsewhere [73, 74]. This patient-administered sexual history taking can be computer or paper-based [75–78]. The use of a computerised tool to elicit patients’ sexual histories has the advantages of saving physicians’ time, of being acceptable to both patients and physicians, of improving care for patients’ specific needs by patient screening and routing, and of improving information collection, especially sensitive information [74, 79, 80]. While high literacy rates [96.1% among males and 92% among females] and a rapid increase in computer/Internet usage in Vietnam [0.3% in 2000 to 34% in 2012] [81] may suggest that ACASI could be feasible for sexual history taking in Vietnam, further research in this area is necessary to evaluate the practicality of using this approach in a country like Vietnam, and whether patients and health professionals would access information on sexual health via e-technology.

Lack of privacy/confidentiality in health facilities in Vietnam, together with the judgmental attitudes of healthcare workers has resulted in delayed treatment for many STI cases [82]. Although patients privacy and confidentiality are stipulated in Vietnamese law [83, 84], elaboration of these are limited in the medical curriculum and current national guidelines. In this regard, placing emphasis on developing a strong culture of patient rights and changing behaviours through simple interventions such as ensuring a closed door and limiting staff movement during consultations can help improving patients’ privacy/confidentiality. The association between lack of privacy/confidentiality and lack of counselling may further compromise patient’s access to comprehensive STI services and this should further investigated.

Another barrier to STI care is lack of counselling. Counselling is important to identify individuals at risk and promote behavioural changes [85–88]. In Vietnam, counselling is mostly provided by physicians to patients as part of the regular physician–patient interaction or in specialised counselling situations, such as during HIV voluntary testing and counselling, prevention of mother-to-child transmission of HIV. However, the quality and delivery of counselling varies among services [89–91]. Physicians’ perceived time pressure may also require consideration. For instance, other healthcare professionals, like nurses and midwives, could assume responsibility for patient counselling to alleviate physician time constraints [92–94]. However, these allied health staff should be adequately trained on counselling before they are assigned for this new responsibility. Additionally, counselling can be included in both undergraduate and post-graduation medical education programs along with CME programs in order to ensure that the physicians have the skills necessary to providing most appropriate counselling. In this study, the low proportion of respondents mentioning lack of counselling should be investigated more deeply in further studies since as this may suggest the limited awareness of physicians of what constitutes appropriate and relevant sexual health counselling.

Finally, amongst the key barriers to STI care reported by physicians in the present study, two out of every ten respondents mentioned lack of reimbursement. Lack of reimbursement has been identified as a barrier for physicians to screen patients for HIV [37, 66] and to adhere to medical guidelines [38] and that financial incentive has been reported to be effective in changing physicians’ practice [95], also that financial incentive together with reminder and feedback may be more effective than incentive only [96]. A survey in Vietnam reported that salary levels and lack of incentives were the highest cause of job dissatisfaction in healthcare [60]. While it can be hypothesized that improvement in physicians’ reimbursement can lead to improvements in STI services, it is necessary to investigate the cost-effectiveness of this approach versus other approaches. For example, further research might examine whether providing higher remuneration to doctors from poor districts would result in higher quality STI care delivery and better STI management.

The limitations of this study need to be acknowledged. Self-reported responses can be subject to social desirability bias with the respondents choosing the responses which they regard as desirable rather than reporting their actual practices [97]. However, the use of surveys for eliciting information from healthcare providers is a common research methodology [54, 98–100]. In our study, the respondents were briefed by one of the researchers on the nature of the survey including its purpose and methods, the assurances of the confidentiality of the respondents’ information, and their right to participate or withdraw from the survey. During the briefing, the respondent physicians were made aware of the confidentiality of study results that no personal identifiable would be reported. It was expected that this could reduce bias. Another concern with the study relates to the representativeness of the sample. Our sample of physicians contained a higher proportion of assistant doctors and females than the national average. In Vietnam, there were 7.2 doctors and 6.22 assistant doctors per 100,000 population [101]. Also, our sample did not include physicians in private practice and had a higher representation of D&V doctors in the sample. However, it is believed that most physicians in Vietnam were in dual practices, who work both in the government and private sector [102, 103]. This suggests non-representativeness of the sample in relation to the health workforce profile in Vietnam. Therefore, caution is needed in interpreting and generalising the findings of this study to all regions of Vietnam. This study was limited in its geographic scope by funding and other resource constraints. Another limitation of the study is that the list of barriers was pre-defined based on available literature. Although an “Other, please specify” response was made available, physicians’ responses might be subject to priming effect where respondents’ responses may be influenced by the response categories already listed [104]. Although the respondents were instructed to tick the response/s that applied to them, the response category “No barrier” was not available which could lead to bias in responses. Future research studies may benefit from a more exhaustive list of barriers or an open-ended question where the respondents are required to list the barriers themselves. Furthermore, a behavioural model incorporating perceived barriers should be used in the development of questionnaire as well as interpretation of findings [105, 106]. Future studies should be carried out on a much larger nationally representative sample of physicians to provide better representation of issues in the management of STIs in Vietnam.

## Conclusions

This study has identified several barriers to STI care in medical practice in Vietnam. The findings are discussed in the context of evidence from other research studies as well as programmatic issues in health service provision in Vietnam. Results of the study can be used to improve areas in STI care including policy and practice implications.
